# Handgrip strength and muscle quality in Australian women: cross‐sectional data from the Geelong Osteoporosis Study

**DOI:** 10.1002/jcsm.12544

**Published:** 2020-02-15

**Authors:** Sophia X. Sui, Kara L. Holloway‐Kew, Natalie K. Hyde, Lana J. Williams, Monica C. Tembo, Mohammadreza Mohebbi, Marlene Gojanovic, Sarah Leach, Julie A. Pasco

**Affiliations:** ^1^ School of Medicine Deakin University Geelong VIC Australia; ^2^ Biostatistics Unit, Faculty of Health Deakin University Geelong VIC Australia; ^3^ GMHBA Geelong VIC Australia; ^4^ Department of Medicine–Western Campus The University of Melbourne St Albans VIC Australia; ^5^ Department of Epidemiology and Preventive Medicine Monash University Melbourne VIC Australia; ^6^ Barwon Health University Hospital Geelong Geelong VIC Australia

**Keywords:** Handgrip strength, Muscle strength, Muscle quality, Normative data: Population‐based study, Women

## Abstract

**Background:**

Low handgrip strength (HGS) is a measure of poor skeletal muscle performance and a marker of ill health and frailty. Muscle quality (MQ) is a measure of muscle strength relative to muscle mass. We aimed to develop normative data for HGS and MQ, report age‐related prevalence of low HGS and MQ, and determine the relationship with age, anthropometry, and body composition for women in Australia.

**Methods:**

This cross‐sectional analysis included data from 792 women (ages 28–95 years) assessed by the Geelong Osteoporosis Study. Duplicate measures of HGS were performed for each hand with a dynamometer (Jamar) and the mean of maximum values used for analyses. Dual energy X‐ray absorptiometry‐derived lean mass for the arms was used to calculate MQ as HGS/lean mass (kg/kg). Body mass index (BMI) was categorized as normal (BMI < 25.0 kg/m^2^), overweight (25.0–29.9 kg/m^2^), and obese (>30.0 kg/m^2^). Fat mass index (FMI) was calculated as whole body fat/height^2^ (kg/m^2^) and appendicular lean mass index (ALMI) as lean mass of arms and legs/height^2^ (kg/m^2^).

**Results:**

Mean (±SD) of HGS values for normal BMI, overweight, and obese groups were 25 (±7), 24 (±7), and 24 (±7) kg, *P* = 0.09, and for MQ, 12 (±3), 11 (±3), and 10 (±3) kg/kg, *P* < 0.001. Our data indicated a quadratic relationship between age and HGS or MQ. Mean HGS and MQ remained stable until the fifth age decade then declined steadily with increasing age; therefore, we used data for women (*n* = 283) aged 28–49 years as the young adult reference group, with mean (SD) values for HGS 28 (±6) kg and MQ 12 (±3) kg/kg. The prevalence of low (*T*‐score < −2) HGS and MQ for women 80 years and older was 52.2% and 39.6%, respectively. In multivariable models, age‐adjusted HGS was associated with FMI (*B* = −0.13, *P* = 0.004) and ALMI (1.03, <0.001) while age‐adjusted MQ was associated with BMI (−0.15, <0.001) but not with FMI. In a sensitivity analysis, the same pattern remained after the removal of 129 women who reported hand and/or arm pain.

**Conclusions:**

Mean HGS and MQ declined with advancing age in older women. Our data suggest that while mean HGS increased with appendicular lean mass and decreased with body fat mass, there was no association with BMI. By contrast, MQ decreased with increasing BMI, but not with increasing adiposity.

## Introduction

As skeletal muscle is required for movement, muscle deterioration results in physical weakness and poor mobility. Evaluating the strength and quality of skeletal muscle is important in the assessment of poor muscle performance, which is a key contributor to sarcopenia, frailty, and loss of independence in elderly populations.^1^, ^2^, ^3^, ^4^ There are reported differences in handgrip strength (HGS) according to age, sex, socio‐demographic factors, and anthropometry, including height and body mass index (BMI).^5^, ^6^, ^7^ Muscle quality (MQ), which expresses muscle strength relative to muscle mass, also declines with age, and marked inter‐individual differences in rates of loss have been reported.^8^, ^9^, ^10^, ^11^ Similar to previous studies,^8^, ^12^, ^13^ we define upper extremity MQ as the ratio of HGS to arm lean mass. Additionally, MQ related to appendicular lean mass (ALM) was assessed, as indicated in another study.^14^

There are few published normative data for HGS and MQ in the Australian population^15^ and employing such data derived from other populations may bias estimates of muscle performance in this population.^7^ The aim of this study was to develop normative data for HGS and MQ for a sample of Australian women, report age‐related prevalence of low HGS and MQ, and describe how HGS and MQ vary with age, anthropometry, and body composition.

## Methods

### Study design and participants

This cross‐sectional analysis was conducted as part of the Geelong Osteoporosis Study, which is a population‐based, prospective cohort study; further detailed information about the Geelong Osteoporosis Study is published elsewhere.^16^ Briefly, participants were randomly selected using electoral rolls for the Barwon Statistical Division, surrounding Geelong in south‐eastern Australia. At baseline (1993–1997), an age‐stratified sample of 1494 women was enrolled, with a 77% response, and, in 2005, this sample was supplemented with further 246 women aged 20–29 years using the same sampling method. Participants in this cohort are mostly Caucasian (~99%). This analysis utilized data from the 15 year follow‐up, conducted from 2010 to 2014. Of 848 potential participants who returned for this follow‐up, 792 women aged 28–95 years provided complete data for these analyses involving HGS, and of these, 751 provided data on lean mass. The study was approved by the Barwon Health Human Research Ethics Committee. Written, informed consent was obtained from all participants.

### Measures

#### Characteristics

Weight and height were measured to the nearest ±0.1 kg and ±0.001 m, respectively. BMI was calculated as weight/height^2^ and categorized as normal (BMI < 25.0 kg/m^2^), overweight (BMI 25.0–29.9 kg/m^2^), or obese (BMI > 30.0 kg/m^2^), according to criteria from the World Health Organization.^17^ Body fat mass, arm lean mass, and ALM (lean mass of the arms and legs) were obtained from whole body dual energy X‐ray absorptiometry (Lunar Prodigy‐Pro, Madison, WI, USA). Short‐term precision (calculated as the coefficient of variation on repeated whole body scans) was 1.7% for whole body fat mass, 1.9% for arm lean mass, and 0.9% for ALM. Fat mass index (FMI) was calculated as whole body fat mass/height^2^ (kg/m^2^) and ALM index (ALMI) as ALM/height^2^ (kg/m^2^). Arm and/or hand pain was documented using a 10‐point scale ranging from ‘no pain’ (0) to ‘as severe as I can imagine’ (10). Arm and/or hand pain was recognized if severity of score was ≥4.^18^

#### Handgrip strength

Handgrip strength was measured using a hand‐held dynamometer (Jamar, Sammons Preston, Bolingbrook, IL, UK). Trained researchers explained and demonstrated the testing procedure to each participant before measurement trials. With the participant seated in a comfortable position and the arm holding the dynamometer flexed at the elbow to 90°, the participant was asked to squeeze the device as hard as possible for several seconds, and the peak reading was recorded. This procedure was duplicated for each hand, and the participant was asked which hand was dominant. There was no time interval between trials. The maximum value for each hand was used for analysis, and the overall HGS represented the mean of maximum values. For descriptive purposes, maximum HGS has been reported separately for the right and left hand and for the dominant and non‐dominant hand. Overall HGS has been used in all statistical analyses and referred to as HGS unless otherwise indicated.

#### Muscle quality

Upper extremity MQ, defined as the ratio of muscle strength (kg) to lean mass (kg),^8^ was calculated as overall HGS/mean of lean mass from both arms.^8^ MQ calculated this way has been used in regression analysis unless otherwise indicated. Additionally, MQ was determined as HGS in relation to ALM (kg/kg).^14^

### Statistical analysis

The distribution of continuous data was checked visually using histograms and Quantile–Quantile plots. Intergroup differences were tested using *t*‐tests or ANOVA for continuous parametric data, Mann–Whitney or Kruskal–Wallis for continuous non‐parametric data, and *χ* ^*2*^ test for categorical data. The young adult reference group was identified as women aged 28–49 years. *T*‐scores for HGS and MQ were calculated as the number of standard deviations (SDs) from the reference means. Prevalence of low HGS and MQ corresponded to *T*‐scores < −2. Regression modelling revealed a non‐linear (quadratic) association between age and HGS or MQ. Spline curves were also fitted and compared with the quadratic regression plots as a model of goodness of fit measure for adequateness of a quadratic model. Multivariable models were developed to identify the best predictors for HGS and MQ by considering age (centred), anthropometry, and measures of body composition. In a sensitivity analysis, modelling was repeated after excluding women with hand and/or arm pain.

We also compared our HGS data with another population‐based Australian study conducted in South Australia, which excluded participants with arm and/or hand pain.^15^ We used general linear models to report estimated mean and 95% confidence intervals (CIs) for our HGS data in each hand, stratified by age decades (excluding participants with arm and/or hand pain). We then observed whether mean HGS from the South Australian study fell within the 95% CIs of our data. In a separate analysis, the median and interquartile range (IQR) values of overall HGS from our study were compared with data from Africa, China, Europe/North America, the Middle East, South America, South Asia, and South East Asia^7^ using boxplots. Minitab (v18, USA) and Stata (v10 Stata Corporation LP, College Station, TX, USA) were used to perform the statistical analyses.

## Results

*Table* 1 presents the participant characteristics. Of 792 participants, 726 (91.6%) reported right hand dominance. Mean HGS was greater in the dominant compared with the non‐dominant hand and in the right versus the left hand (both *P* < 0.001). A similar pattern was observed for MQ (both *P* < 0.001). Participants ranged in age from 28 to 95 years, and mean BMI was in the overweight range.

**Table 1:** Characteristics for 792 participants

Anthropometry and demographics	
Age (year)	56.5 (42.6–69.8)
Weight (kg)	74.1 (±16.1)
ALM (kg)	17.8 (2.7)
Height (cm)	162.0 (±6.5)
BMI (kg/m^2^)	28.2 (±5.9)
ALMI (kg/m^2^)	6.8 (0.9)
FMI (kg/m^2^)	11.9 (4.7)

HGS (kg)	
Dominant hand	25 (±7)
Non‐dominant hand	23 (±7)
Right hand	25 (±7)
Left hand	24 (±7)
Overall	24 (±7)

Arm lean mass (kg)	
Dominant arm	2.3 (±0.4)
Non‐dominant arm	2.3 (±0.4)
Right arm	2.3 (±0.4)
Left arm	2.3 (±0.4)
Overall	2.3 (±0.3)

MQ (HGS/arm lean mass, kg/kg)	
Dominant hand	11 (±3)
Non‐dominant hand	11 (±3)
Right hand	11 (±3)
Left hand	11 (±3)
Overall	11 (±3)

MQ (HGS/ALM, kg/kg)	
Dominant hand	1.4 (0.4)
Non‐dominant hand	1.3 (0.4)
Right hand	1.4 (0.4)
Left hand	1.3 (0.4)
Overall	1.4 (0.3)
	
Pain	
Arm or hand pain	129 (16.5%)

Data are displayed as median (interquartile range), mean (±standard deviation) or *n* (%).

ALM, appendicular lean mass (kg); ALMI, appendicular lean mass index (kg/m^2^); BMI, body mass index (kg/m^2^); FMI, fat mass index (kg/m^2^); HGS, handgrip strength (kg); MQ, muscle quality (kg/kg).

### Young adult reference group and *T*‐scores

Our data indicate that HGS and MQ remained stable until the end of the fifth decade and declined with age thereafter. Accordingly, 283 women aged 28–49 years comprised the young adult reference group. The prevalence estimates of women with *T*‐scores < −2 in each age decade for HGS and MQ are listed in *Table* 2. Cut‐off points equivalent to *T*‐scores of both −2.0 and −1.0 for HGS and MQ are shown in *Table* 3.

**Table 2:** Handgrip strength and muscle quality for participants stratified by age groups

	Age (year)	*n*	Dominant hand	Non‐dominant hand	Overall	*T*‐score < −2 *n* (%)
HGS (kg)						
	<30	24	28 (±7)	26 (±8)	27 (±7)	1 (4.2)
	30–39	134	29 (±7)	27 (±7)	28 (±6)	2 (1.5)
	40–49	125	29 (±6)	28 (±6)	29 (±6)	1 (0.8)
	50–59	151	26 (±6)	25 (±7)	25 (±6)	7 (4.6)
	60–69	157	23 (±6)	23 (±6)	23 (±5)	14 (8.9)
	70–79	132	21 (±6)	20 (±6)	21 (±6)	21 (15.9)
	≥80	69	16 (±6)	15 (±5)	15 (±5)	36 (52.2)
MQ (HGS/arm lean mass, kg/kg)						
	<30	21	13 (±3)	11 (±3)	12 (±3)	1 (4.8)
	30–39	130	12 (±3)	11 (±3)	12 (±3)	4 (3.1)
	40–49	125	13 (±3)	12 (±3)	13 (±2)	1 (0.8)
	50–59	151	12 (±3)	11 (±3)	11 (±3)	7 (4.6)
	60–69	152	11 (±3)	10 (±3)	11 (±3)	10 (6.6)
	70–79	124	10 (±3)	9 (±3)	10 (±3)	13 (10.5)
	≥80	48	9 (±4)	8 (±3)	8 (±3)	19 (39.6)
MQ (HGS/ALM, kg/kg)						
	< 30	21	1.5 (±0.3)	1.4 (±0.4)	1.4 (±0.3)	1 (4.8)
	30–39	130	1.5 (±0.3)	1.4 (±0.3)	1.4 (±0.3)	8 (6.2)
	40–49	124	1.6 (±0.3)	1.5 (±0.3)	1.6 (±0.3)	1 (0.8)
	50–59	151	1.5 (±0.3)	0.4 (±0.4)	1.4 (±0.3)	9 (6.0)
	60–69	152	1.4 (±0.3)	1.3 (±0.3)	1.3 (±0.3)	9 (5.9)
	70–79	124	1.3 (±0.4)	1.2 (±0.3)	1.3 (±0.3)	13 (10.5)
	≥80	48	1.1 (±0.4)	1.0 (±0.3)	1.0 (±0.4)	16 (33.3)

Data are shown as mean (± SD). Prevalence of low handgrip strength and muscle quality (*T*‐score < ‐2) are also shown, expressed as *n* (%).

ALM, appendicular lean mass (kg); HGS, handgrip strength; MQ, muscle quality.

**Table 3:** Young adult reference data (28–49 years) for handgrip strength (kg; *n* = 283) and muscle quality (kg/kg; *n* = 276) and cut points equivalent to *T*‐scores of −1.0 and −2.0

Variable	Category	Mean (SD)	* *T* score = −1.0	* *T* score =−2.0
HGS (kg)				
	Dominant hand	29 (±6)	23	16
	Non‐dominant hand	27 (±7)	20	14
	Overall	28 (±6)	22	16
				
MQ (HGS/arm lean mass, kg/kg)				
	Dominant hand	13 (±3)	10	8
	Non‐dominant hand	12 (±3)	9	7
	Overall	12 (±3)	10	7
				
MQ (HGS/ALM, kg/kg)				
	Dominant hand	1.6 (0.3)	1.3	1.0
	Non‐dominant hand	1.4 (0.3)	1.1	0.8
	Overall	1.5 (0.3)	1.2	0.9
				

* *T*‐score = −1.0, hand grip strength and muscle quality 1SD below the young adult reference mean; ^*^ *T*‐score = −2.0, hand grip strength and muscle quality equivalent to 2SD below the young adult reference mean.

ALM, appendicular lean mass (kg); HGS, handgrip strength; MQ, muscle quality; SD, standard deviation.

### Hangrip strength, muscle quality, age, anthropometry, and body composition

**Figure 1:**
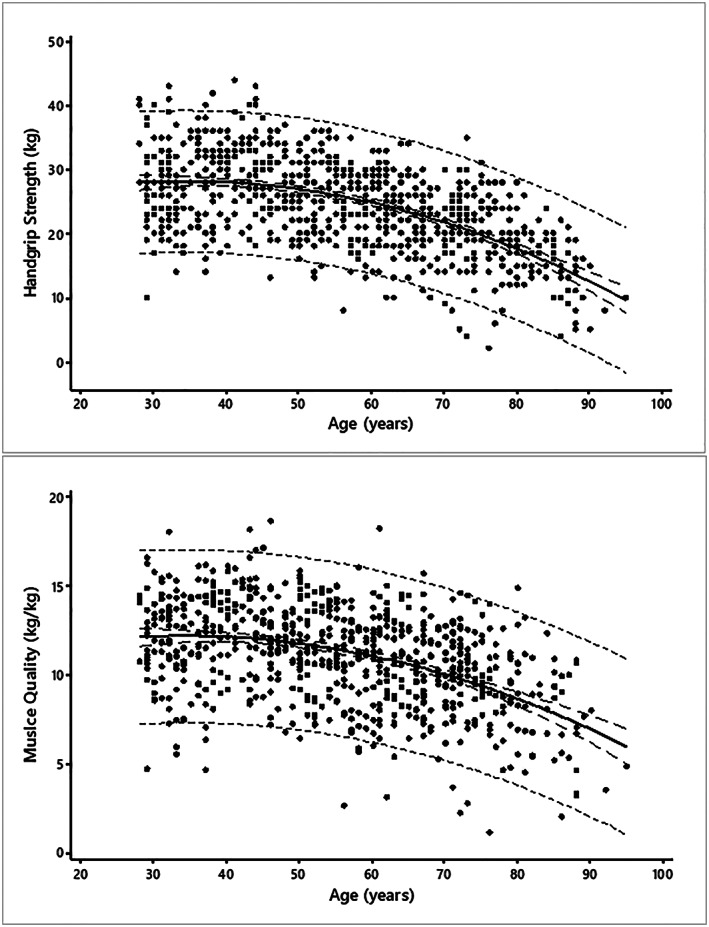
The association between age and (A) handgrip strength and (B) muscle quality. Shown are the regression line (continuous line), 95% prediction interval (short dashes) and 95% confident interval (long dashes).

*Table* 2 shows mean (±SD) values for HGS and MQ stratified by age decade. There was a non‐linear age‐related decline in HGS (*Figure* 1A; *Table* 4). A separate model revealed an apparent negative and marginal association between HGS and BMI (*B* = ±0.08, *P* = 0.05); however, this association was lost after adjusting for age (*P* = 0.21). HGS was negatively associated with FMI (*B* = −0.26, *P* < 0.001) and positively with ALMI (*B* = +1.96, *P* < 0.001). These associations were sustained after adjusting for age (Models 2 and 3), and the best model (Model 4) showed that FMI and ALMI predicted HGS independent of age (*Table* 4).

**Table 4:** Models for predicting handgrip strength and muscle quality

Category	Model	Variables	Coefficient (*B*)	*SE*	*P*	*R* ^*2*^ adjusted
HGS (kg)	1					32.4%
		Age_c_^*^	−0.22	0.01	<0.001	
		(Age_c_)^2*^	−0.005	0.001	<0.001	
		Constant	25.66	0.28	<0.001	
	2					33.6%
		Age_c_^*^	−0.22	0.01	<0.001	
		(Age_c_)^2*^	−0.005	0.001	<0.001	
		FMI	−0.16	0.05	0.001	
		Constant	27.50	0.61	<0.001	
	3					31.0%
		Age_c_^*^	−0.20	0.01	<0.001	
		(Age_c_)^2*^	−0.005	0.001	<0.001	
		ALMI	1.08	0.25	<0.001	
		Constant	18.38	1.69	<0.001	
	4					33.8%
		Age_c_^*^	−0.21	0.01	<0.001	
		(Age_c_)^2*^	−0.005	0.001	<0.001	
		FMI	−0.13	0.05	0.004	
		ALMI	1.03	0.25	<0.001	
		Constant	20.24	1.82	<0.001	
MQ (kg/kg)	1					19.8%
		Age_c_^*^	−0.07	0.006	<0.001	
		(Age_c_)^2*^	−0.002	0.0004	<0.001	
		Constant	11.34	0.13	<0.001	
	2					29.7%
		Age_c_^*^	−0.07	0.005	<0.001	
		(Age_c_)^2*^	−0.002	0.0003	<0.001	
		BMI	−0.15	0.02	<0.001	
		Constant	15.74	0.44	<0.001	

ALM, appendicular lean mass (kg); ALMI, appendicular lean mass index (kg/m^2^); BMI, body mass index (kg/m^2^); FMI, fat mass index (kg/m^2^); HGS, handgrip strength (kg); MQ, muscle quality (kg/kg).^*^Age centred around mean age (56.5 years).

There was also a non‐linear age‐related decline in MQ (*Figure* 1B; *Table* 4). MQ was negatively associated with BMI (*B* = −0.16, *P* < 0.001, Adjusted *R* ^*2*^ = 0.11), and this association was sustained after adjusting for age (*Table* 4). MQ was negatively associated with FMI (*B* = −0.07, *P* = 0.004); however, this association (*P* = 0.21) was lost after adjusting for age.

### Sensitivity analysis

After excluding 129 women who reported pain in the hand and/or arm, mean HGS and MQ values increased slightly, but the patterns of age‐associated changes in HGS and MQ were preserved (*Table* A5).

### Comparison of our data with other studies

We found that mean HGS values from the study in South Australia were within 95% CI of our data (Table A6) except for the fourth age decade (both hands).

**Figure 2:**
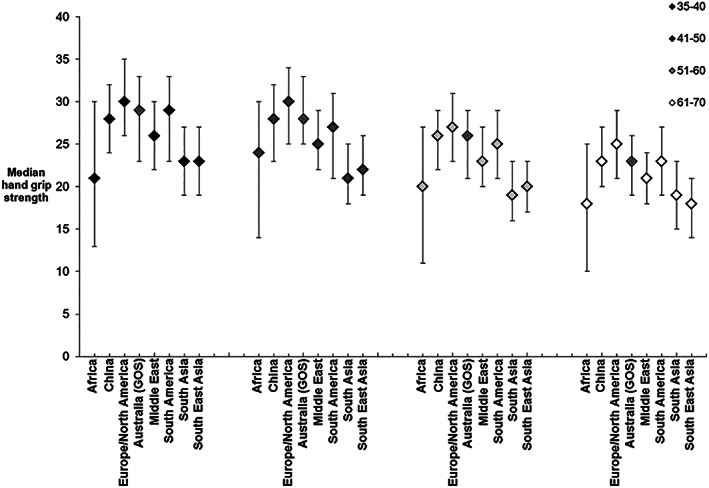
Comparison of handgrip strength data from our Australian study (the Geelong Osteoporosis Study) with data reported from other (international) studies. Data are shown as medians and interquartile ranges.

A comparison of the age‐specific median (IQR) of HGS values from our participants with values reported from studies from other geographical locations is shown in *Figure* 2. For each age group, the point estimates for HGS from this study fell within the IQRs reported for other studies, with exception of those from South Asia and South East Asia, where the median values were lower.

## Discussion

We report normative data for HGS and MQ for women from south‐eastern Australia and provide young reference data for both parameters. In addition, we report how HGS and MQ vary with age, anthropometry, and measures of body composition. Our data are comparable with population‐based data reported from other populations of women in Australia and overseas.^7^, ^15^

We found that HGS in women peaked in the fifth age decade (at a mean of 29 kg). This is consistent with normative data from UK, in an analysis that combined data from 12 studies to report that female HGS peaked at an average of 31 kg around the fourth decade of life (26–42 years).^5^

We identified that HGS remained essentially stable during the fourth and fifth age decades, followed by a subsequent decrease with advancing age. This was consistent with a meta‐analysis of studies investigating muscle strength and age in the general population, where the age‐related decline in HGS was 0.06 kg/year from age 20 to 50 years, and the decline increased thereafter by 0.37 kg/year, also suggesting a change point at about age 50 years.^19^ The rapid decline in HGS after age 50 years is likely to be explained by declining oestrogen levels that accompany menopause.^19^

A study in South Australia, conducted in 2012, reported normative data for HGS in a community‐based sample of 3206 of men and women (1315 women) recruited randomly from the telephone directory.^15^ There were minor inconsistencies between their data and ours that may have been due to differences in recruitment strategies and inclusion/exclusion criteria, as their participants with arm or hand pain and/or arthritis lasting more than 1 month were excluded. To our knowledge, there are no other reported normative data for HGS in Australia.

A recent study by Leong *et al*. included HGS measures for 125 462 healthy adults, to develop a HGS reference range for healthy adults across a wide range of diverse geographic regions in 21 countries in Europe, America, Asia, the Middle East, and Africa, and stratified the data by age, sex, BMI, ethnicity, and geographic region.^7^ No Australian data were included in this study. We found that our data were consistent with measures reported for women from the other geographical locations, as our age‐specific point estimates fell within IQRs reported from other geographical locations, with the exception of South Asia and South East Asia.

Although there have been several reports in the literature relating to MQ,^8^, ^10^, ^11^, ^12^, ^14^ there have been inconsistent ways of quantifying MQ. Across the life span, MQ in the arms is greater than MQ in the legs.^20^ Most studies have measured MQ for upper and lower extremes separately. For example, in studies by Newman *et al*.^12^ and Hairi *et al,*,^8^ MQ for the upper extremity was derived from the ratio of HGS to arm lean mass, and for lower extremity, MQ was defined as the ratio of quadriceps strength to leg lean mass.^8^, ^12^ However, in another study, MQ referred to HGS in relation to ALM (kg/kg).^14^ We have provided normative data for both HGS/arm lean mass and HGS/ALM.

A longitudinal study of women aged 20.2–92.2 years in the USA reported a linear age‐related decline in MQ at the arms.^20^ Here, we describe a non‐linear (quadratic) decline of MQ with age, albeit less pronounced than the decline in HGS. This pattern was observed in our data, because the age‐related decline in muscle strength exceeded the decline in muscle mass.^21^

In order to identify threshold for low HGS and MQ, we have calculated *T*‐scores using the young adult reference data. Our *T*‐score of −2.0 for HGS (16 kg) is comparable with a cut‐off value of 16.4 kg derived from a Korean sample of 2556 women aged 19–80 years, defined as maximal HGS of the dominant hand and measured using a digital hand dynamometer; the cut‐off values were documented as quintile points, and low HGS was defined as the lowest 20%.^22^ A Japanese study suggested a cut‐off value of 18.2 kg for low HGS for a sample of 2468 healthy women aged 65 years and over (mean age 71.9 ± 5.5 years), corresponding to the lowest 20% of this older population.^23^

To our knowledge, this is the first study to report *T*‐scores for MQ. There is debate that MQ may be a suitable parameter for identifying pre‐sarcopenia, defined as a condition characterized by low muscle mass without associated decline of muscle strength or muscle function.^24^ Further studies are needed to validate MQ as an assessment tool.

Our data suggest that HGS was not associated with BMI. In contrast, the previously described multinational study by Leong *et al*. suggested a positive association between HGS and BMI (categorized as underweight, healthy weight, overweight, and obese), although this relationship appeared to plateau for BMI values in the obese category.^7^ An Australian study by Massy‐Westropp *et al*. also demonstrated a positive, albeit weak, relationship between BMI and the HGS in the youngest (20–30 years) and oldest age (70 + years) groups in their sample; however, a higher BMI was negatively related to HGS in other age groups.^15^

In our study, HGS was positively associated with lean mass and negatively with fat mass after adjusting for age, while age and BMI (but not FMI) contributed to the best model for predicting MQ. The Health Ageing and Body Composition Study, which aimed to examine whether lower muscle mass and higher fat mass have independent effects on loss of muscle strength and MQ in older men and women, found that lower extremity strength was associated with fat mass and leg strength after adjusting for leg lean mass and MQ; age and body fat were inversely associated with MQ in older adults.^12^ A study of 321 individuals (215 women) aged 50 years and older reported that MQ was associated with physical function and BMI, but found no age‐related decline in MQ.^10^

We recognize several strengths and weaknesses in our study. A major strength is that we assessed women drawn from the general population using a random sampling technique, so they were not selected on the basis of disease. Furthermore, objective measures of lean mass and HGS were obtained according to standard protocols. However, although participants were encouraged to produce maximal HGS, we cannot exclude the possibility of suboptimal effort. In contrast to other studies that included adults from age 20 years, our youngest participant was aged 28 years. However, an age‐related decline in HGS was not evident until age 50 years, and so, this truncated age range in the reference sample is unlikely to impact on the estimated cut‐off points for low HGS. Lastly, as this study included women only and most were Caucasian, our findings may not be generalizable to other populations.

## Conclusions

In conclusion, the overall pattern of decline in HGS and MQ with advancing age was non‐linear. Our young adult reference data are useful for identifying low HGS and MQ in relation to population distributions, in alignment with the approach recommended by European Working Group on Sarcopenia (EWGSOP [2]and EWGSOP2 [3]). Our data provide normative data for HGS and MQ, which may be a useful for assessing poor muscle performance, sarcopenia, or frailty in women.

## Conflict of interest

Sophia X. Sui, Kara L. Holloway‐Kew, Natalie K. Hyde, Lana J. Williams, Monica C. Tembo, Mohammadreza Mohebbi, Marlene Gojanovic, Sarah Leach, and Julie A. Pasco declare that no competing interests exist.

## Funding

The Geelong Osteoporosis Study was funded by the National Health and Medical Research Council (NHMRC) Australia (projects 251638, 628582). SXS was supported by a Deakin Postgraduate Scholarship in conjunction with GMHBA. KLH‐K was supported by an Alfred Deakin Postdoctoral Research Fellowship; NKH was supported by a Dean's Research Postdoctoral Fellowship (Deakin University) and LJW by an NHMRC Career Development Fellowship (1064272). MCT was supported by Deakin Postgraduate Scholarship. The funding organizations played no role in the design or conduct of the study, in the collection, management, analysis and interpretation of the data, nor in the preparation, review and approval of the manuscript.

## Ethical approval

All procedures performed in studies involving human participants were in accordance with the ethical standards of the institutional and national research committees and with the 1964 Helsinki declaration and its later amendments or comparable ethical standards. The study was approved by the Human Research Ethics Committee at Barwon Health.

## Informed consent

Written, informed consent was obtained from all participants included in the study.

## Appendix

**Table A1:** Sensitivity analysis excluding women who reported hand pain and/or arm pain

Age (year)	Handgrip strength (HGS, kg), *n* = 652		
	Dominant hand	Non‐dominant hand	Overall
<30	28 (±7)	26 (±7)	27 (±7)
30–39	29 (±7)	27 (±7)	28 (±6)
40–49	29 (±6)	28 (±7)	28 (±6)
50‐59	27 (±5)	25 (±6)	26 (±5)
60–69	25 (±5)	23 (±5)	24 (±5)
70‐79	22 (±6)	21 (±6)	21 (±6)
≥80	17 (±5)	15 (±4)	16 (±4)
All	26 (±7)	24 (±7)	25 (±7)
	Muscle quality (MQ, kg/kg), *n* = 620		
	Dominant hand	Non‐dominant hand	Overall
<30	13 (±3)	12 (±3)	12 (±3)
30–39	12 (±3)	12 (±3)	12 (±3)
40–49	13 (±2)	12 (±3)	12 (±2)
50–59	12 (±2)	11 (±3)	12 (±2)
60–69	11(±2)	11 (±3)	11 (±2)
70–79	10 (±3)	10 (±3)	10 (±3)
≥80	9 (±3)	8 (±2)	9 (±3)
All	12 (±3)	11 (±3)	11 (±3)

Data are shown as mean (±SD).

**Table A2:** Comparison of Geelong Osteoporosis Study (GOS) data and South Australian data^15^ for handgrip strength

Age (year)	Geelong (*n* = 652)				South Australia (*n* = 1315)	
	Right hand	95% CI	Left hand	95% CI	Right hand	Left hand
<30	28	(25‐30)	26	(24‐29)	30 (7)	28 (6.1)
30‐39	29	(28‐30)	27	(26‐28)	31 (6.4)	29 (6)
40‐49	29	(28‐30)	28	(27‐29)	29 (5.7)	28(5.7)
50‐59	27	(26‐28)	26	(25‐27)	28 (6.3)	26 (5.7)
60‐69	24	(23‐25)	23	(22‐25)	24 (5.3)	23 (5)
70+	20	(19‐21)	19	(18‐20)	20 (5.8)	19 (5.5)
All	26	(25‐27)	25	(24‐25)	/	/

Data (GOS) are shown as mean and 95% confident interval.

CI, confidence interval.
